# Modelling analysis of dietary behaviors and oral health status to assess the impact on the mental well-being of elderly individuals: a cross-sectional survey study

**DOI:** 10.3389/fnut.2025.1486987

**Published:** 2025-03-19

**Authors:** Chan Huang, Mingzhu Song, Xiao Wei, Xingyan Wang, Honglin Dai, Zhiqiong Gou, Feiyang Chenwu, Yanqiu Jiang, Jie Wan, Yurun Guo, Xiaoping Yu

**Affiliations:** ^1^School of Preclinical Medicine & School of Nursing, Chengdu University, Chengdu, China; ^2^Department of Respiratory and Thoracic Surgery, West China Tianfu Hospital, Sichuan University, Chengdu, China

**Keywords:** oral health, eating behavior, elderly, mental health, modelling analysis

## Abstract

**Background and objective:**

Global aging is becoming a pressing concern, with a heightened focus on the mental well-being of the elderly population. The mental health of the elderly is influenced by a plethora of factors such as physical health, social support, lifestyle, and psychological aspects. This study investigates the influence of oral health and eating behaviors on depression, anxiety, and dementia in elderly individuals aged 65 and older in China. The aim is to determine the effect sizes of these factors and establish a foundation for implementing tailored intervention strategies.

**Study design and methods:**

The cross-sectional survey study employed data from the 2020 follow-up of the Chinese Longitudinal Healthy Longevity Survey (CLHLS) released in April 2020. The study included a cohort of 3,188 eligible older adults. Seven aspects of oral health were assessed, encompassing the evaluation of the number of teeth, dental restorations, tooth brushing habits, occurrence of tooth pain, as well as cheek or jaw pain. Additionally, 17 items pertaining to eating behaviors were examined, covering dietary aspects such as staple foods, vegetables, fruits, tastes, and the use of cooking oils. The study assessed depression and sleep through 11 items, with higher scores reflecting a predisposition toward depression. A scoring threshold of over 27 identified individuals in the group prone to depression. Anxiety levels were assessed through seven items, where higher scores denoted a predisposition toward anxiety. Participants scoring above 0 were categorized into the anxiety-prone group. Cognitive function was assessed through seven items, with higher scores suggestive of a propensity toward dementia. Participants with scores exceeding 8 were categorized into the dementia-prone group. Rank-sum tests and chi-square tests were employed for the univariate analysis of variations in depression, anxiety, and dementia among elderly individuals with varying oral health and eating behaviors. Variables demonstrating statistical significance in the univariate analysis were further examined in logistic regression analysis.

**Results:**

This study uncovered a correlation between the oral health and dietary behaviors of middle-aged and elderly individuals and their vulnerability to depression, anxiety, and dementia. Our findings showed that about 7.62% of middle-aged and elderly individuals in China were prone to depression. Meanwhile, around 49% of this population had inadequate teeth to chew and digest properly, and nearly 10% lacked dental restorations. Elderly individuals who had dental surgery had a 50% lower risk of depression compared to those who did not (OR = 0.58). Additionally, 36.7% of this group were prone to anxiety, and 7.53% were at risk of developing dementia. Elderly individuals who brushed their teeth at least twice a day and maintained oral hygiene were less likely to develop anxiety and dementia, with odds ratios of 0.72 and 0.78, respectively. This study conducted in China revealed that among individuals aged 65 and older, 22% reported experiencing tooth pain, while 11% reported experiencing cheek or jaw pain. Controlling tooth, cheek, or jaw pain significantly diminished the likelihood of anxiety in elderly population, with odds ratios of 0.79 and 0.69, respectively. The study also uncovered that consuming an adequate amount of fresh vegetables on a daily basis was highly advantageous in preserving the mental well-being of elderly individuals, and might reduce the risk of depression by 32.5%, anxiety by 50.3%, and dementia by 50%. Elderly individuals could potentially prevent anxiety and dementia by consuming an adequate amount of fruits daily. Conversely, a diet high in salt and spice was potentially associated with an increased risk of anxiety in this population. Furthermore, middle-aged elderly individuals (under 80) exhibited a potentially higher susceptibility to anxiety compared to older elderly individuals (aged 80 and above).

**Conclusion:**

An immediate imperative exists to enhance oral health education, elevate oral hygiene standards, and guarantee prompt dental restoration among middle-aged and elderly populations in order to mitigate their susceptibility to depression, anxiety, and dementia. Safeguarding the mental health of elderly individuals necessitates the coordination of diverse disciplines, encompassing dentistry, nutrition, and public health expertise.

## 1 Introduction

The global challenge of aging is growing more pressing by the day. According to United Nations data, it is projected that by 2050, the global population aged 65 and older will reach 1.6 billion, accounting for 16% of the total global population (1). China, being the most populous country globally, is also grappling with considerable aging. According to the data from China’s National Bureau of Statistics, it is projected that by 2023, the population aged 65 and older in China will surpass 260 million, accounting for 18.7% of the total population (2). This rapid growth in the elderly population presents substantial challenges to the social, economic, and healthcare systems.

The prevalence of mental health concerns among the elderly is garnering growing recognition in scholarly and clinical spheres. Globally, depression is prevalent among the elderly, as indicated by World Health Organization (WHO) statistics which report a depression prevalence rate of approximately 7 to 8% among this demographic (3). In China, the rate of depression among the elderly is significantly high. Recent research have indicated that approximately 10 to 15% of Chinese individuals aged 65 and above experience depression (4). Anxiety is a significant mental health concern prevalent among the elderly. International studies have shown that approximately 10 to 15% of the elderly population experience severe symptoms of anxiety (5). In China, the prevalence of anxiety among the elderly has been rising in recent years, and the National survey data has suggested that approximately 12 to 14% of elderly individuals have exhibited symptoms of anxiety. Dementia is another significant mental health concern that profoundly impacts the quality of life in the elderly population. According to global data, about 5 to 8% of the elderly individuals aged 65 and older are affected by dementia (6). Likewise, the prevalence of dementia in China is also increasing, and it is projected that by 2030, the number of dementia patients in China will exceed 10 million, placing a substantial burden on families and society (7).

Recent studies have shown that the mental health of the elderly individuals is influenced by a multitude of factors, such as physical health, social support, lifestyle choices, and psychological well-being (8). Physical health can be a contributing factor to the development of mental health issues, specifically depression and anxiety, as individuals dealing with chronic diseases and functional decline are at a heightened risk (9). Lack of social support and social isolation has also been identified as significant factors influencing the mental well-being of older adults (10). Psychological factors, such as life satisfaction and self-efficacy, also play crucial roles in the psychological well-being of older adult (11). Recent research has suggested that oral health and eating behavior of the elderly may also be significant factors that can impact their mental well-being. Oral health issues, such as tooth loss and oral diseases, are intricately linked to the mental health status of the elderly population. Studies have shown that older adults with inadequate oral health are more likely to display symptoms of depression and anxiety (12). Specifically, oral issues like tooth loss and periodontal disease can result in reduced chewing function, impacting dietary intake and overall well-being, potentially worsening symptoms of depression (13). Furthermore, oral health problems among the elderly can adversely affect their engagement in social activities, leading to heightened feelings of loneliness and exacerbating depressive symptoms (14, 15).

Oral health and eating behavior are intricately connected, with increasing attention being given to research on the correlation between eating patterns and the mental well-being of older adults. Some studies have indicated a potential relationship between inadequate dietary choices, malnutrition, and the manifestation of depression and anxiety symptoms in older adults (16, 17). Recent studies have suggested that specific nutrients in the diet, such as Omega-3 fatty acids and vitamin B12, are significantly associated with cognitive function and emotional well-being in elderly individuals (18). Several studies have investigated the relationship between oral health and cognition (19), but the literature on this topic is still limited, necessitating additional research and validation (20).

This study aims to investigate the influence of oral health and eating behavior on the mental health of Chinese elderly aged 65 and older, focusing on the factors that contribute to depression, anxiety, and cognitive impairment. This study not only enriches the theoretical research on factors influencing the mental health of the elderly but also offers scientific evidence for policymakers and healthcare professionals, aiding in the development of targeted interventions to enhance the quality of life for older adults.

## 2 Methods

### 2.1 Study subjects and sampling methods

The data for this study were derived from the Chinese Longitudinal Healthy Longevity Survey (CLHLS), an elderly tracking survey project conducted by the Center for Healthy Aging and Development Studies / National School of Development at Peking University. The CLHLS survey covered 23 provinces, municipalities, and autonomous regions in China, focusing on individuals aged 60 and above, as well as their offspring. All participants volunteered to participate in the study and provided written informed consent. In cases where elderly participants were unable to sign the forms themselves, their family members signed on their behalf. The survey has been approved by the Biomedical Ethics Committee of Peking University (IRB00001052-13074). Baseline surveys were conducted in 1998, followed by subsequent surveys in 2000, 2002, 2005, 2008–2009, 2011–2012, 2014, and 2017–2018, comprising a total of 113,000 home visits (21). This study utilized the most recent data from the follow-up survey conducted in April 2020, which covered the period 2017–2018 and includes 15,874 individuals aged 60 and above.

**Figure 1:**
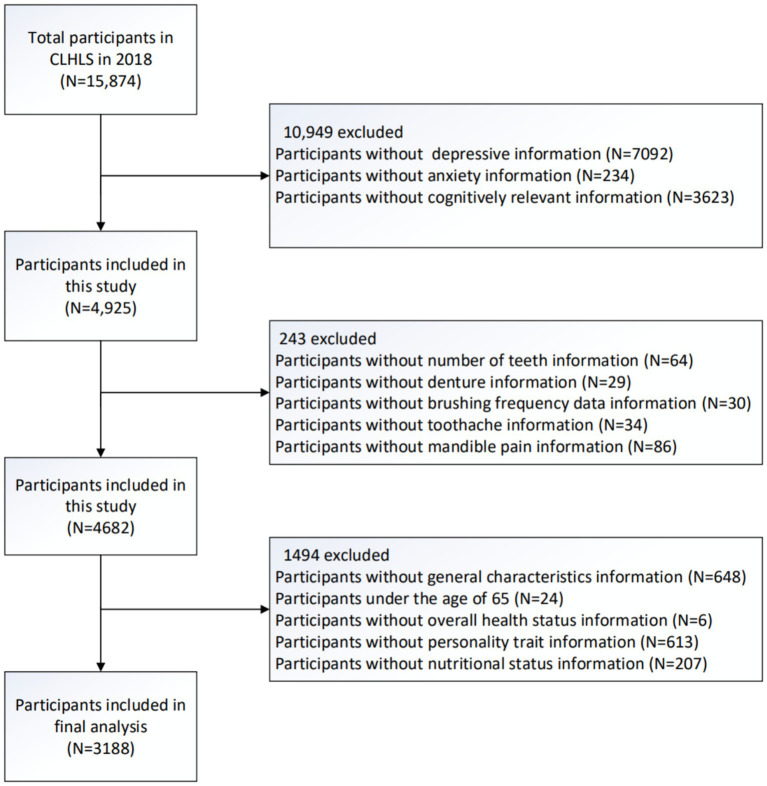
Flow chart of research object selection.

In our study, individuals aged 65 and above were included who completed assessments regarding their oral health and mental well-being. Exclusion criteria involved individuals under 65 years of age and those with missing data on variables related to oral health, mental health, and general characteristics. The study subjects were selected according to the process illustrated in Figure 1, and a final cohort of 3,188 eligible elderly individuals were obtained.

### 2.2 Measurement indicators

#### 2.2.1 Independent variables

This study includes independent variables categorized into two parts. (1) Oral health status, in the Supplementary Information Questionnaire-1 (SIQ-1), (2) Diet and nutrition: This comprises 17 items, including: staple food, cooking oil, intake of fresh fruits, intake of fresh vegetables, taste preference, in the (SIQ-2), meats, fish and other aquatic products, eggs, bean products, pickled vegetables or kimchi, white sugar or candies, garlic (garlic sprouts/garlic yellow/garlic sprouts/green garlic, etc.), dairy products (milk/formula/yogurt/ice cream, etc.), nuts (peanuts/walnuts/chestnuts/sunflower seeds, etc.), fungi and algae (mushrooms/wood ear/white fungus/kelp/seaweed, etc.), vitamins (A/C/E/calcium tablets, etc.) or health supplements, and medicinal plants (Ginseng/ Astragalus /Wolfberry/Angelica, etc.). The survey assessed the intake of 12 types of food using a five-point scale, in the (SIQ-3).

#### 2.2.2 Dependent variables

In this study, the dependent variables assessed are the mental health of older adults, evaluated using three subscales for depression, anxiety, and cognitive function. Respondents were asked to report on their experiences over the previous 1 to 2 weeks. (1) Depression scale: includes 11 items, in the (SIQ-4). The first nine entries are assigned to the first dimension depression score. The nine items mentioned above are rated on a five-point scale ranging from 1 to 5 (1 = Always, 2 = Often, 3 = Sometimes, 4 = Rarely, 5 = Never). Higher scores on this scale indicate a higher level of depression severity. Items 1, 2, 3, 4, 6, 8, and 9 are reverse-coded, and the depression score is obtained by summing the responses to these nine items. Scores exceeding 27 are categorized as indicative of a propensity for depression. The second dimension pertains to sleep evaluation, encompassing both the quality and duration of daily sleep. (2) Anxiety scale: includes 7 items, in the (SIQ-5). The seven items mentioned above are rated on a four-point scale ranging from 0 to 3 (0 = None, 1 = Several days, 2 = More than half the days, 3 = Almost every day). Higher scores on this scale signify increased levels of anxiety. Elderly individuals with scores above 0 are categorized as prone to anxiety. (3) Cognitive function: evaluated through the use of the Community Screening Instrument for Dementia (CSI-D), which comprises seven items, in the (SIQ-6). Each item is scored on its correctness, with one point for a perfect answer and two points for an incorrect answer. The total score is calculated by adding scores from all seven items, with higher scores indicating lower cognitive status. Individuals who score higher than 8 points on the CSID scale are classified as dementia-prone.

#### 2.2.3 Control variables

Two groups of control variables are set in this study, (1) General characteristics: includes six items: age, gender, ethnicity, living situation, smoking, drinking, in the (SIQ-7). (2) Basic status: includes three items: living conditions, health status, changes in health status over the past year, in the (SIQ-8).

### 2.3 Statistical methods

After preliminary exploration of the data, it was found that the elderly mental health score presented a skewed distribution. Therefore, the rank sum test was used to compare the differences in the mental health of the elderly with different demographic characteristics, and the chi-square test was used to compare the elderly with different oral states. Whether there was any difference in the susceptibility rate of depression, anxiety and dementia, the chi-square test was used to analyze the relationship between oral state and eating behavior, and the statistically significant variables in univariate analysis were included in multivariate analysis. Logistic regression was used to analyze the independent effect of each factor on the mental health of the elderly. The above statistical analysis used STATA SE 17.0 (Copyright 1985–2025 Stata Corp LLC Stata Corp 4,905 Lakeway Drive College station, Texas 77,845 USA), with a test level of 0.05.

## 3 Results

### 3.1 Analysis of mental health among elderly population with different demographic characteristics

The study included 3,188 elderly individuals aged between 65 and 115 years. In the sample, there were 179 individuals who reached the age of 100 or older, with the group aged over 80 years comprising 43.5% of the total sample size. This research analyzed the relationship between demographic characteristics and the mental health of the elderly. As shown in Table 1, the results of the univariate analysis revealed that age, gender, living arrangement, smoking, and drinking significantly influenced depression scores (*p* < 0.05). Elderly individuals aged 80 and above, women, those living in nursing homes, non-smokers, and non-drinkers tended to have relatively higher depression scores. Age, gender, and drinking significantly impacted anxiety scores (*p* < 0.05), and those under 80, women, and non-drinkers comparatively elevated levels of anxiety. Age, gender, and smoking exhibited a significant influence on cognitive scores (*p* < 0.05), and individuals aged 80 and above, women, and non-smokers exhibited relatively superior cognitive performance.

**Table 1:** Mental health analysis of the elderly with different demographic characteristics.

Demographic characteristics		N (%)	Depression scores		Anxiety scores		Cognitive scores		Sleep quality		Sleep duration	
			Mean rank	*p*	Mean rank	*p*	Mean rank	*p*	Mean rank	*p*	Mean rank	*p*
Age (year)	<80	1800 (56.46)	1,562	0.023*	1,644	0.0001**	1,526	<0.001**	1,617	0.097	1,521	<0.001**
	≥80	1,388 (43.54)	1,637		1,530		1,683		1,565		1,690	
Gender	Male	1708 (53.58)	1,528	<0.001**	1,528	<0.001**	1,567	<0.001**	1,485	<0.001**	1,682	<0.001**
	Female	1,480 (46.42)	1,671		1,671		1,626		1721		1,494	
Ethnicity	Han	3,042 (95.42)	1,587	0.049	1,597	0.446	1,595	0.749	1,588	0.047*	1,591	0.367
	Minority nationality	146 (4.58)	1741		1,546		1,584		1737		1,660	
Lifestyle of the elderly	At home	2,578 (80.87)	1,555	<0.001**	1,583	0.177	1,594	0.982	1,571	<0.001**	1,621	<0.001**
	In community	504 (15.81)	1737		1,641		1,602		1,664		1,501	
	In a nursing home	106 (3.32)	1851		1707		1,597		1873		1,338	
Smoking	Yes	579 (18.16)	1,516	0.024*	1,561	0.265	1,557	0.019*	1,473	<0.001**	1,649	0.109
	No	2,609 (81.84)	1,612		1,602		1,603		1,621		1,582	
Drinking	Yes	535 (16.78)	1,402	<0.001**	1,512	0.009**	1,582	0.438	1,507	0.012*	1,657	0.079
	No	2,653 (83.22)	1,633		1,611		1,597		1,612		1,582	

* *p* < 0.05, statistically significant; ** *p* < 0.01, statistically significant.

Additionally, we examined the sleep patterns of elderly individuals with varying characteristics, revealing significant impacts on sleep quality from factors such as gender, living arrangements, smoking, and drinking (*p* < 0.05). In this study, it was found that women, ethnic minorities, individuals in institutional care, non-smokers, and non-drinkers exhibited relatively higher levels of sleep quality. Gender, age, and living arrangements had a significant impact on sleep duration (*p* < 0.05), and men, individuals aged 80 and above, and those receiving home care exhibited longer sleep durations.

### 3.2 Relationship between oral health and mental health in the elderly

Among the 3,188 elderly individuals included in the study, 680 had complete tooth loss, while 894 had fewer than 10 teeth, indicating that nearly 50% of the participants experienced impaired chewing function. According to the threshold values outlined in the part of methods, we identified elderly individuals who are susceptible to depression, anxiety, and dementia. Statistical analysis showed that 7.62% of the participants exhibited susceptibility to depression, 36.7% to anxiety, and 7.53% to dementia. In Table 2, the distribution of susceptibility to depression, anxiety, and dementia among elderly population with different oral health conditions was given. Univariate analysis revealed a marked disparity in the susceptibility to depression between elderly individuals who did not use dentures and those who did, with rates of 9.3 and 5.4%, respectively. Moreover, individuals who brushed their teeth rarely or occasionally demonstrated a relatively higher susceptibility to depression (9.97%), whereas those who brushed their teeth daily exhibited a lower susceptibility (5.66%). Elderly individuals experiencing toothache, cheek, or jaw pain exhibited a significantly increased vulnerability to depression, with rates of 10.25 and 12.57%, respectively.

**Table 2:** Analysis of depression, anxiety and dementia susceptibility in the elderly with different oral conditions.

Oral situation		*N* (%)	Depression susceptibility *n* (%)		Anxiety susceptibility *n* (%)		Dementia susceptibility *n* (%)	
Missing tooth condition	Complete absence	680 (21.33)	54 (7.94)	*χ*^2^ = 0.449 *p* = 0.799	218 (32.06)	χ^2^ = 8.864 *p* = 0.012*	91 (13.38)	χ^2^ = 61.970 *p* < 0.001**
	<10 teeth	894 (28.04)	71 (7.94)		350 (39.15)		81 (9.06)	
	≥10 teeth	1,614 (50.63)	118 (7.31)		602 (37.30)		68 (4.21)	
Denture use status?	Yes	1,370 (42.97)	74 (5.40)	χ^2^ = 16.828 *p* < 0.001^*^	463 (33.80)	χ^2^ = 8.724*p* = 0.003**	104 (7.59)	χ^2^ = 0.014 *p* = 0.907
	No	1818 (57.03)	169 (9.30)		707 (38.89)		136 (7.48)	
Brush frequency	Rarely or occasionally	632 (19.82)	63 (9.97)	χ^2^ = 13.793 *p* = 0.001^**^	266 (42.09)	χ^2^ = 10.126 *p* = 0.006**	93 (14.72)	χ^2^ = 64.127 *p* < 0.001**
	Once a day	1,342 (42.1)	76 (5.66)		481 (35.84)		93 (6.93)	
	Two or more times a day	1,214 (38.08)	104 (8.57)		423 (34.84)		54 (4.45)	
Do you have a toothache?	Yes	712 (22.33)	73 (10.25)	χ^2^ = 0.449 *p* = 0.799	336 (47.19)	χ^2^ = 43.431 *p* < 0.001**	48 (6.74)	χ^2^ = 0.815 *p* = 0.367
	No	2,476 (77.67)	170 (6.87)		834 (33.68)		192 (7.75)	
Pain in the cheek or jaw	Yes	350 (10.98)	44 (12.57)	χ^2^ = 9.009 *p* = 0.003^**^	183 (52.29)	χ^2^ = 41.110 *p* < 0.001**	31 (8.86)	χ^2^ = 0.997 *p* = 0.318
	No	2,838 (89.02)	199 (7.01)		987 (34.78)		209 (7.36)	

* *p* < 0.05, statistically significant; ** *p* < 0.01, statistically significant.

In addition, it was also shown that non-denture users had a significantly higher susceptibility to anxiety than denture users (38.89% vs. 33.80%) in Table 2. Those who rarely or occasionally brushed their teeth demonstrated a comparatively higher susceptibility to anxiety (42.09%), while those who brushed their teeth twice daily showed a lower susceptibility rate (34.84%). Significantly higher percentages of elderly individuals experiencing toothache, cheek, or jaw pain showed susceptibility to anxiety, with rates of 47.19 and 52.29%, respectively. The data also revealed a lack of significant difference in susceptibility to dementia between individuals who used denture users and those who did not (7.48% VS. 7.59%). However, those who rarely or occasionally brushed their teeth exhibited a greater vulnerability to dementia (14.72%), while those who brushed twice daily showed a reduced susceptibility rate (4.45%). These univariate analyses did not consider confounding factors, and further multivariate analysis is needed to determine the independent effects of these factors.

### 3.3 Relationship between eating behavior and mental health in the elderly

Among the 3,188 elderly individuals, the majority consumed rice as their staple food, while only a small proportion followed a mixed diet. In China, 70% of the elderly population consumes fresh vegetables daily, while fewer than 30% consume fresh fruit on a daily basis. We further analyzed the correlation between food consumption and mental health in the elderly. Table 3 depicted the results of a study investigating the influence of various dietary habits on the mental well-being of the elderly, analyzing the effects of staple food types, fresh fruits, fresh vegetables, types of cooking oil, and taste preferences. The univariate analysis revealed that individuals who consumed rice, pasta, or mixed grains, ate fresh fruits and vegetables daily or almost daily, and had a preference for spicy foods, exhibited a relatively lower susceptibility to depression (4.4, 4.9, 5.2, 6.0%, respectively, *p* < 0.001). Similarly, the analysis showed that individuals who incorporated fresh fruits and vegetables into their daily diet exhibited reduced susceptibility to anxiety (27.1, 32.4%, *p* < 0.001). Although the influence of staple food and taste on anxiety was statistically significant, it approached the test threshold, suggesting the need for further investigation. Additionally, the analysis of factors influencing susceptibility to dementia indicated that daily consumption of fresh fruits and vegetables along with the use of vegetable oil significantly reduced susceptibility to dementia (3.9, 6.1, 7.1%, *p* < 0.01). However, these findings from the univariate analyses may be affected by confounding factors, highlighting the necessity of conducting additional multivariate analyses to ascertain independent effects.

**Table 3:** Analysis of depression, anxiety and dementia susceptibility of the elderly with different dietary habits.

Dietary habit		*N* (%)	Depression susceptibility, *n* (%)		Anxiety susceptibility, *n* (%)		Dementia susceptibility, *n* (%)	
The type of carbohydrate	Rice	1934 (60.66)	174 (9.0)	χ^2^ = 16.630 *p* < 0.001**	745 (38.5)	χ^2^ = 7.118 *p* = 0.028*	143 (7.4)	χ^2^ = 0.173 *p* = 0.917
	Noodles or bread	465 (14.59)	34 (7.3)		155 (33.3)		35 (7.5)	
	Mix rice and grain	789 (24.75)	35 (4.4)		270 (34.2)		62 (7.9)	
Frequency of eating fruits	Eat it every day	851 (26.69)	42 (4.9)	χ^2^ = 19.222 *p* < 0.001**	231 (27.1)	χ^2^ = 58.495 *p* = <0.001**	33 (3.9)	χ^2^ = 33.344 *p* = <0.001**
	Frequent eating	772 (24.22)	61 (7.9)		272 (35.2)		51 (6.6)	
	Occasionally eat	908 (28.48)	68 (7.5)		395 (43.5)		82 (9.0)	
	Rarely or never	657 (20.61)	72 (11.0)		272 (41.4)		74 (11.3)	
Frequency of eating vegetables	Eat it every day	2,233 (70.04)	116 (5.2)	χ^2^ = 2.242 *p* = 0.134	723 (32.4)	χ^2^ = 1.457 *p* = 0.227	137 (6.1)	χ^2^ = 9.055 *p* = 0.003**
	Frequent eating	702 (22.02)	82 (11.7)		309 (44.0)		63 (9.0)	
	Occasionally eat	191 (5.99)	32 (16.8)		106 (55.5)		27 (14.1)	
	Rarely or never	62 (1.94)	13 (21.0)		32 (51.6)		13 (21.0)	
Types of cooking oil	Vegetable oil	2,916 (91.47)	216 (7.4)	χ^2^ = 73.411 *p* < 0.001**	1,061 (36.4)	χ^2^ = 69.120 *p* < 0.001**	207 (7.1)	χ^2^ = 36.399 *p* = <0.001**
	Animal oil	272 (8.53)	27 (9.9)		109 (40.1)		33 (12.1)	
Taste preference	Insipidity	2,137 (67.03)	136 (6.4)	χ^2^ = 23.07 *p* < 0.001**	753 (35.2)	χ^2^ = 11.850 *p* = 0.019*	165 (7.7)	χ^2^ = 4.381 *p* = 0.357
	Salty	659 (20.67)	60 (9.1)		260 (39.5)		52 (7.9)	
	Sweet	150 (4.71)	21 (14.0)		57 (38.0)		9 (6.0)	
	Pungent	84 (2.63)	5 (6.0)		43 (51.2)		8 (9.5)	
	Indeterminate taste	158 (4.96)	21 (13.3)		57 (36.1)		6 (3.8)	

* *p* < 0.05, statistically significant; ** *p* < 0.01, statistically significant.

### 3.4 Relationship between oral health and eating behavior

Differences in food intake among elderly individuals with varying oral health conditions were further examined, and the results were outlined in Table 4, which included an analysis of the intake of fresh fruits and vegetables and 12 other food categories, such as meat, fish, and eggs. Significant statistical variances were observed in the intake distribution (mean ranks) of most food items between elderly individuals with uncorrected tooth loss and those with corrected tooth loss or functional teeth, with the exception of beans. Elderly individuals with uncorrected tooth loss exhibited lower consumption of various foods, but showed a relatively higher intake of sugar or candy.

**Table 4:** Analysis of different kinds of food intake in the elderly with different oral conditions.

Food type	The tooth is missing and unrepaired^a^, *n* = 634		The missing tooth has been repaired^b^, *n* = 940		The teeth function well^c^, *n* = 1,184		H	*p*
	Sum of ranks (10^4^)	Mean Rank (10^4^)	Sum of ranks (10^4^)	Mean Rank (10^4^)	Sum of ranks (10^4^)	Mean Rank (10^4^)		
Fresh fruits	76	0.120	135	0.144	169	0.143	45.608	<0.001^**^
Fresh vegetables	82	0.129	132	0.140	167	0.141	16.636	<0.001^**^
Meat	84	0.133	125	0.133	171	0.144	15.532	<0.001^**^
Fish and other aquatic products	77	0.122	131	0.139	173	0.146	40.381	<0.001^**^
Eggs	84	0.132	134	0.143	163	0.138	7.308	0.026^*^
Soybean products	84	0.132	131	0.139	166	0.140	4.696	0.096
Preserved vegetables or infuse vegetables	83	0.131	134	0.143	164	0.139	8.115	0.017^*^
White sugar or candies	92	0.145	133	0.141	156	0.132	15.294	<0.001^**^
Garlic	81	0.128	133	0.141	166	0.140	13.805	0.001^**^
Dairy products	80	0.127	134	0.143	166	0.140	19.726	<0.001^**^
Nuts	74	0.116	133	0.141	173	0.146	68.014	<0.001^**^
Fungi and algae	76	0.120	132	0.140	172	0.145	49.621	<0.001^**^
Vitamins or health supplements	83	0.131	128	0.136	170	0.144	24.713	<0.001^**^
Medicinal plants	82	0.129	128	0.136	171	0.144	33.204	<0.001^**^

a Completely missing teeth or missing more than 10 teeth, no dentures and other restorations.

b Completely missing teeth or missing more than 10 teeth, have been fitted with dentures and other repairs.

c Missing less than 10 teeth does not affect chewing function. *p < 0.05, statistically significant; **p < 0.01, statistically significant.

### 3.5 Multivariate analysis of mental health in the elderly

In order to account for potential confounding effects, this study incorporated variables that yielded statistically significant resulted from the univariate analyses into a multivariate analysis. The analysis also controlled for living conditions, health status, and changes in health status by including them as covariates in a multivariate logistic regression model.

Model 1, shown in Table 5, demonstrated that, after accounting for confounding variables, significant predictors of depression included oral health status and consumption of fresh vegetables. Conversely, other oral health-related factors such as tooth brushing, toothache, cheek, or jaw pain did not exhibit statistically significant in the multivariate analysis. Likewise, dietary behaviors like staple food consumption, fresh fruit intake, and taste preferences did not show significance. This suggested that the repair status of missing teeth and the daily intake of fresh vegetables were independent factors and significantly affected the risk of depression, separating from living conditions, health status, and changes in health status. The findings revealed that elderly individuals with repaired missing teeth had a 50% lower risk of depression compared to those with unrepaired missing teeth (OR = 0.58). Furthermore, as the frequency of fresh vegetable intake decreased, the risk of depression increased, with individuals who rarely or never consumed fresh vegetables having a threefold higher risk of depression than those who had them daily (OR = 3.07).

**Table 5:** Multivariate analysis of depression susceptibility in the elderly (Model 1).

Influencing factor	OR (95% CI)	SE	*Z*	*p*
Dental condition				
The tooth is missing and unrepaired				
The missing tooth has been repaired	0.58 (0.37, 0.91)	0.13	−2.39	0.017
The teeth function well	0.80 (0.53, 1.21)	0.17	−1.04	0.296
Frequency of eating vegetables				
Eat it every day/almost every day				
Frequent eating	2.18 (1.47, 3.22)	0.43	3.90	<0.001
Occasionally eat	2.34 (1.36, 4.00)	0.64	3.09	0.002
Rarely or never	3.07 (1.33, 7.12)	1.32	2.62	0.009
Living condition				
Good	2.08 (1.67, 2.59)	0.23	6.52	<0.001
General health status				
Good	2.68 (2.11, 3.40)	0.33	8.04	<0.001
Healthy change				
Better	1.45 (1.15, 1.82)	0.17	3.12	0.002
*p*	<0.001			
R^2^	0.268			

Only statistically significant variables are shown in the table. The control variables were age, gender, retirement style, smoking, drinking, life status, general health status and health change.

Model 2, as presented in Table 6, revealed that, following adjustment for confounding variables, distinct factors significantly associated with anxiety encompassed tooth brushing, toothache, cheek or jaw pain, fresh vegetable and fruit intake, and taste preferences. These six variables emerged as independent factors that significantly impacted the risk of experiencing anxiety, distinct from factors such as living conditions, health status, changes in health status, age, and gender. The findings demonstrated that individuals who brush their teeth two or more times daily experienced a 28% reduction in anxiety risk. Addressing toothache was linked to a 21% decrease in anxiety risk, while managing cheek or jaw pain was associated with a 31% reduction in anxiety risk.

**Table 6:** Multifactor analysis of anxiety susceptibility in the elderly (Model 2).

Influencing factor	OR (95% CI)	SE	*Z*	*p*
Brushing frequency				
Rarely brush or brush occasionally				
Once a day	0.79 (0.63, 1.00)	0.09	−1.96	0.050
Two or more times a day	0.72 (0.56, 0.93)	0.09	−2.53	0.011
Do you have a toothache?	0.79 (0.63, 0.99)	0.09	−2.06	0.040
Yes				
Pain in the cheek or jaw	0.69 (0.52, 0.93)	0.10	−2.46	0.014
Yes				
Frequency of eating vegetables				
Eat it every day/almost every day				
Frequent eating	1.56 (1.26, 1.93)	0.17	4.12	<0.001
Occasionally eat	2.02 (1.44, 2.83)	0.35	4.08	<0.001
Rarely or never	1.99 (1.12, 3.54)	0.59	2.33	0.009
Frequency of eating fruits				
Eat it every day/almost every day				
Frequent eating	1.11 (0.85, 1.45)	0.15	0.76	0.446
Occasionally eat	1.56 (1.21, 1.99)	0.20	3.48	<0.001
Rarely or never	1.19 (0.91, 1.56)	0.16	1.26	0.207
Taste preference				
Insipidity				
Salty	1.24 (1.01, 1.52)	0.13	2.03	0.042
Sweet	0.98 (0.66, 1.46)	0.20	−0.08	0.936
Pungent	1.97 (1.22, 3.20)	0.49	2.75	0.006
Indeterminate taste	0.81 (0.55, 1.19)	0.16	−1.07	0.284
Living condition				
Good	1.17 (1.04, 1.32)	0.07	2.68	0.007
General health status				
Good	1.53 (1.37, 1.71)	0.09	7.42	<0.001
Healthy change				
Better	1.10 (0.98, 1.23)	0.06	1.63	0.103
Age (year)				
<80	0.79 (0.66, 0.95)	0.07	−2.52	0.012
Gender				
Male	1.37 (1.15, 1.62)	0.12	3.50	<0.001
*p*	<0.001			
R^2^	0.087			

Only statistically significant variables are shown in the table. The control variables were age, sex, alcohol consumption, life status, general health status, and health change.

Moreover, elderly individuals who have infrequent fresh vegetable consumption faced nearly twice the anxiety risk compared to those consuming fresh vegetables almost daily. Similarly, individuals who only occasionally eat fresh fruit had a 1.56 times higher anxiety risk than those eating fresh fruit nearly every day. Furthermore, elderly individuals with a heavy salt diet exhibited a 1.24 times greater anxiety risk compared to those with a light diet, and those with a spicy diet were at a 1.97 times higher risk for anxiety than those with a light diet.

Model 3, displayed in Table 7, indicated that significant predictors of dementia included tooth brushing and fresh vegetable and fruit intake, even after controlling for confounding factors. Other variables related to oral health, such as oral health status, toothache, and cheek or jaw pain, did not show statistical significance in the multivariate analysis. Similarly, variables concerning dietary behavior, such as staple food and taste preferences, were not found to be significant predictors. These findings suggested that the frequency of tooth brushing and daily intake of fresh vegetables and fruits were independent factors that significantly affected the risk of dementia, regardless of factors like cooking oil types, living conditions, health status, changes in health status, and gender. The study results demonstrated that brushing teeth twice daily or more experienced a 22% reduction in dementia risk. Elderly individuals who eat fresh fruit only occasionally had a 1.65 times higher risk of dementia compared to those who eat fresh fruit almost daily. Those who do not eat fresh vegetables had twice the risk of dementia compared to those who eat fresh vegetables almost daily (OR = 2.00).

**Table 7:** Multivariate analysis of dementia susceptibility in the elderly (Model 3).

Influencing factor	OR (95% CI)	SE	*Z*	*p*
Brushing frequency				
Rarely brush or brush occasionally				
Once a day	0.86 (0.69, 1.08)	0.10	−1.30	0.193
Two or more times a day	0.78 (0.61, 1.00)	0.10	−2.00	0.046
Frequency of eating fruits				
Eat it every day/almost every day				
Frequent eating	1.12 (0.86, 1.47)	0.15	0.87	0.387
Occasionally eat	1.65 (1.29, 2.11)	0.21	4.03	<0.001
Rarely or never	1.28 (0.98, 1.67)	0.17	1.82	0.069
Frequency of eating vegetables				
Eat it every day/almost every day				
Frequent eating	1.52 (1.23, 1.87)	0.16	3.93	<0.001
Occasionally eat	1.95 (1.39, 2.72)	0.33	3.91	<0.001
Rarely or never	2.00 (1.13, 3.54)	0.58	2.38	0.017
Living condition				
Good	1.16 (1.04, 1.31)	0.07	2.54	0.011
General health status				
Good	1.57 (1.40, 1.75)	0.09	7.96	<0.001
Gender				
Male	1.41 (1.19, 1.69)	0.13	3.87	<0.001
*p*	<0.001			
R^2^	0.077			

Only statistically significant variables are shown in the table. The control variables were age, sex, alcohol consumption, life status, general health status, and health change.

## 4 Discussion

This study examined the mental health factors in Chinese individuals aged 65 and above, with a specific focus on oral health and dietary behavior. The aim is to identify susceptibility factors for depression, anxiety, and dementia among the elderly population, enabling early screening and targeted prevention and management of mental health issues.

In the study, it was discovered that 7.62% of middle-aged and older adults in China were prone to depression, and addressing missing teeth plays a vital role in reducing this susceptibility. Particularly worrisome is the oral health status of this population, as 21% of individuals aged 65 and above are completely edentulous, and 28% have less than 10 teeth. These statistics contrast with earlier findings, which reported edentulous rates of 7% for Chinese elderly individuals aged 65–74 and 8.9% for those aged 50 and above (22). According to the WHO, the global edentulous rate for adults aged 60 and above is estimated to be 23% in 2023 (23). In comparison, the edentulous rates in the United States are 15% among individuals aged 65–74 and 22% among individuals aged 75 and above (24). The rates in other countries are as follows: 22% for Canadian seniors aged 60–79 (25), 19.8% for Hungarian seniors aged 65–74 (26), 20.7% for Spanish seniors (27), 25.5% for Mexican seniors (26), 48% for Turkish seniors (28), 54.7% for Brazilian seniors (29), and 26.9% for French seniors aged 65 and above (30). Generally, adults typically possess between 28 and 32 teeth, comprising incisors, canines, premolars, and molars. A minimum of 20 teeth, including 8 incisors and 12 molars, is essential for adequate chewing function (31). Incisors and canines perform the task of cutting and tearing food, whereas premolars and molars are responsible for grinding and chewing food.

As indicated by this study, almost half (49%) of middle-aged and older adults in China experience challenges in achieving efficient chewing and digestion with their natural teeth, while approximately 10% have unrepaired missing teeth. Numerous studies have documented a range of health risks linked to tooth loss, and addressing this issue through dental restoration can enhance masticatory function, thus enabling elderly individuals to more effectively absorb essential nutrients. Proper nutrition plays a vital role in supporting physical health and ensuring normal brain function. Effective chewing aids in digestion, minimizes gastrointestinal issues, and contributes to overall physical comfort, which in turn fosters a positive outlook. Malnutrition can lead to physical weakness and fatigue, thereby raising the likelihood of depression. Inadequacies in specific vitamins and minerals can significantly influence the proper functioning of the nervous system. For instance, a deficiency in vitamin D can impede the growth and differentiation of nerve cells, while vitamin B6 (pyridoxine) plays a key role in the synthesis and metabolism of various neurotransmitters. Furthermore, vitamin B12 (cobalamin) is essential for the development and upkeep of the nervous system, and insufficient levels can result in nerve damage and various neuropsychiatric symptoms. Iron plays a crucial role in synthesizing hemoglobin, and iron deficiency anemia may lead to insufficient oxygen supply to the brain, affecting the function of nervous system and leading to symptoms like fatigue, lack of concentration, mood swings, and depression. Besides digestive and nutrition-related concerns, the absence of teeth is also associated with osteoporosis, speech disorders, cardiovascular diseases, diabetes, respiratory diseases (32–34), and a shorter lifespan (35).

A study in BMC Public Health highlighted the significant association between tooth loss and indicators of overall health and well-being. It revealed that individuals who were edentulous, particularly the elderly, experienced lower levels of self-rated health, mental well-being, and subjective well-being (36). Consequently, it is advisable for middle-aged and older adults to undergo routine comprehensive oral examinations, promptly engage with dentists, and select suitable restoration procedures tailored to the pattern and position of missing teeth, the condition of the remaining teeth, and individual oral health status. In cases where the remaining natural teeth are healthy, fixed dentures or dental implants may be preferable choices. Alternatively, if oral health is compromised or financial constraints exist, removable dentures can also serve as a viable solution.

It was found that 36.7% of middle-aged and older adults in China were susceptible to anxiety, and 7.53% of people might be at risk of developing dementia. The findings suggested that maintaining good oral hygiene, specifically brushing teeth twice or more daily, might be lower the risk of susceptibility to anxiety and dementia among the elderly. Oral health is intricately linked to overall health. By upkeeping proper oral hygiene practices, such as regular brushing, elderly individuals can decrease the presence of bacteria and microorganisms in the oral cavity, thereby reducing inflammation. Chronic inflammation has been proven to be associated with numerous chronic diseases, including dementia and anxiety. Studies have shown that chronic inflammation may play a role in the development of dementia, including Alzheimer’s disease. Inflammation and infections in the oral cavity have the potential to have the potential to trigger systemic inflammatory responses that affect the brain and expedite cognitive decline (37). Studies conducted in the United States, the United Kingdom, Japan, and other countries have also highlighted that individuals who brush their teeth twice daily have significantly lower chances of developing dementia compared to those who neglect oral hygiene (24, 25). This body of research strongly reinforces the significance of oral hygiene in dementia prevention (38). The survey study discovered that 20% of elderly individuals aged 65 and above in China rarely or never brushed their teeth, whereas 38% brushed their teeth two or more times daily. Elderly individuals who rarely or never brush their teeth exhibited a notably high susceptibility to anxiety (42.09%), while those who brush their teeth twice or more daily demonstrated a relatively lower susceptibility to anxiety (34.84%). This disparity arises from the fact that elderly individuals who neglect oral hygiene are predisposed to oral inflammation, which not only impacts their eating behavior and quality of life but also influences their mental health, potentially leading to increased anxiety levels. For instance, individuals suffering from periodontitis experience a significantly diminished quality of life compared to those with good oral health, influencing their psychological state and emotional well-being (39). Thus, it is crucial for middle-aged and older adults to optimal oral hygiene practices, including using soft-bristled toothbrushes along with fluoride toothpaste, and mastering proper brushing techniques, such as the Bass brushing method, to ensure thorough cleaning of each tooth surface without causing excessive stimulation to the gums. Dentists recommend that individuals in this age group brush their teeth at least twice daily, morning and evening, for a minimum of 2 minutes each session. This routine can effectively reduce the number of bacteria in the mouth, which may reduce the risk of anxiety and dementia. Additionally, it is advisable for those who are able to incorporate mouthwash into their oral care regimen after meals or before bedtime, swishing it around the mouth for approximately 30 s to ensure all areas are properly treated (40).

It also revealed that controlling toothache, cheek, or jaw pain could significantly reduce the risk of anxiety in the elderly. Specifically, 22% of individuals aged 65 and above in China experience toothache, while 11% experience cheek or jaw pain. For those experiencing these types of pain, the susceptibility to anxiety significantly increases to 47.19 and 52.29%, respectively. Toothache, cheek, or jaw pain acts as a potent stimulus that directly influences the mindset and emotions of middle-aged and older adults. This influence triggers stress responses and contributes to feelings of anxiety. Moreover, the presence of pain also hampers normal activities such as eating, talking, and social interactions among the elderly, further impacting their emotional state and mindset. This results in feelings of frustration, dissatisfaction, and irritability. Therefore, it may increase the risk of anxiety susceptibility. Importantly, a study indicated that providing psychological care could significantly reduce pain and effectively alleviate anxiety among the elderly population (41).

Therefore, it is advisable to instruct elderly individuals in relaxation techniques, such as deep breathing and participating in activities of personal interest. Furthermore, effective interventions should also be implemented to manage pain and alleviate anxiety. For instance, rinsing with saline solution can cleanse the affected area and mitigate inflammation to some extent, relieving toothache. A prudent approach is needed when applying an ice pack to the painful tooth, cheek, or jaw area to numb the nerves and reduce pain, although prolonged usage should be avoided to prevent frostbite. Furthermore, chewing on a boiled tea bag can offer relief by leveraging the tannic acid present, aiding in reducing inflammation and fostering healing. In cases where required, medications like ibuprofen or codeine can provide rapid pain relief and alleviate anxiety. Nevertheless, regular oral care practices are imperative in preventing toothache and cheek or jaw pain, encompassing diligent brushing, maintaining oral hygiene, and regular dental check-ups (42).

Eating enough fresh vegetables daily is extremely beneficial for preserving the mental health of elderly individuals by reducing their risk of depression, anxiety, and dementia. It was observed that 70% of Chinese individuals aged 65 and above included fresh vegetables in their daily diet, while only 2% rarely or never did so. Fresh vegetables are a rich source of essential nutrients such as proteins, vitamins (specifically vitamin C and B vitamins), minerals (such as potassium, calcium, and magnesium), and dietary fiber, along with various antioxidants like carotenoids and polyphenols, all of which are vital for the human body. These nutrients play a crucial role in supporting the mental well-being of elderly individuals and reducing their vulnerability to depression, anxiety, and dementia. Studies have demonstrated that folic acid can notably improve cognitive function (43), with green leafy vegetables being a commendable dietary source of this nutrient (44). Moreover, vegetables are also abundant in B vitamins and antioxidants, which can effectively stave off cognitive decline, shield nerve cells from harm, and aid in preserving emotional equilibrium. Consequently, it is recommended to include a sufficient amount of fresh vegetables in your daily diet. Examples of such vegetables include spinach, cabbage, and lettuce, which are rich in folic acid, as well as pumpkins and potatoes, abundant in vitamins and minerals. Carrots and tomatoes are also beneficial choices as they are rich in antioxidants. Consuming a diverse range of vegetables and employing suitable cooking methods to preserve nutrients can significantly enhance cognitive function, promote a positive mood, and reduce the risk of anxiety and depression. Consuming an adequate amount of fresh fruit daily can serve as a protective measure against anxiety and dementia among older adults. Fresh fruits, akin to sufficient intake of fresh vegetables, are rich in various nutrients, such as antioxidants (vitamin C, vitamin E, carotenoids, etc.), B vitamins (particularly folic acid), and essential minerals (potassium, magnesium, calcium, etc.), that may play a role in alleviating anxiety and mitigating the risk of dementia in older individuals. Unlike fresh vegetables, fresh fruits are high in natural sugars, which can trigger the release of hormones like endorphins post-consumption, thus modulating mood and sustaining a positive attitude. Alarmingly, the research showed that while 70% of Chinese individuals aged 65 years and older consumed fresh vegetables daily, fewer than 30% incorporated fresh fruit into their daily diet. Interestingly, there appears to be a disparity in the attention given by the elderly to consuming fresh fruit compared to fresh vegetables. Henceforth, it is advisable for older adults to maintain an appropriate level of fruit consumption tailored to their specific requirements. According to China’s dietary guidelines, a daily intake of 200–350 grams of fruit is suggested (45) http://dg.en.cnsoc.org/article/lsqy.html. When choosing fruits, individual health considerations must be taken into account. For instance, individuals with diabetes should opt for low-sugar fruits, while those with sensitive gastrointestinal tracts ought to avoid overly acidic or cold fruits. Oranges are rich in vitamin C and folate, which are known to enhance cognitive function and alleviate anxiety symptoms. Bananas provide significant amounts of potassium, essential for maintaining proper nervous system functionality and aiding in Alzheimer’s disease prevention. Elderly individuals may vary their fruit choices based on their distinct nutritional demands.

The study revealed a correlation between high-salt and spicy diets and heightened anxiety among the elderly population. Specifically, 21% of Chinese individuals aged 65 and older were found to follow a high-salt diet, while 3% adhered to a spicy diet. Evidence has suggested that prolonged consumption of high levels of salt can increase the risk of cardiovascular diseases (46), ultimately leading to physical discomfort and potential anxiety development. Furthermore, the presence of capsaicin in spicy foods can stimulate the gastrointestinal system in the elderly, compromising digestive function and inducing gastrointestinal discomfort, thereby potentially exacerbating anxiety. Accordingly, it is recommended to regulate salt and spicy food intake, while limiting the consumption of pickled, smoked, and high-salt foods. Choosing low-salt seasonings and opting for easily digestible, light foods like vegetables, fruits, and porridge is advisable. However, it is important not to overly restrict the diet to avoid malnutrition and compromised immunity, which may increase the risk of anxiety. Elderly individuals should also ensure adequate hydration, aiming for a daily intake of at least 1,500 milliliters. Preferred beverages include plain water or diluted tea, while excessive consumption of sugary drinks and caffeinated beverages should be avoided. Adequate hydration facilitates normal metabolism and physiological functions, helps to alleviate physical and psychological stress, and maintains a positive mindset (47).

Moreover, middle-aged elderly individuals in the elderly category (those under 80) exhibited a higher susceptibility to anxiety in comparison to older elderly individuals (aged 80 and above). Middle-aged elderly people may often encounter significant life transitions, such as retirement, children leaving home, and a shrinking social circle, leading to psychological strain and the onset of anxiety. In contrast, older elderly individuals may have already adapted to these life alterations and have developed a decreased sensitivity to external stressors owing to declining health, rendering them less prone to anxiety and more inclined to uphold a positive mindset. So, it is recommended that middle-aged elderly individuals engage in hobbies such as reading, handicrafts, or Tai Chi to enhance life enjoyment and fulfillment, thus reducing feelings of emptiness and anxiety. Additionally, maintaining close contact with family and sharing joys and concerns can also provide significant support in alleviating anxiety (48).

Elderly women had a higher risk of anxiety and dementia compared to men. Post-menopausal women underwent a substantial decrease in estrogen levels, which play a role in safeguarding cholinergic neurons and enhancing cognitive function (49). Consequently, elderly women, with lower estrogen levels, are more prone to cognitive impairments and dementia. Additionally, women generally exhibit greater emotional sensitivity and experience more pronounced fluctuations, rendering them more vulnerable to anxiety and depression. Chronic psychological stress and negative emotions may adversely affect brain function and escalate the risk of dementia. Hence, it is advisable for elderly women to maintain a balanced diet rich in essential nutrients, engage in moderate exercise to enhance brain oxygenation, and cultivate interests and hobbies to enrich their mental well-being and alleviate emotional stress, thereby reducing the likelihood of anxiety (50).

The univariate analysis of the study provided insights into the susceptibility of elderly individuals to depression, anxiety, and dementia. For example, consuming rice, noodles, or grains as staple foods was linked to a reduced risk of depression, and a preference for spicy food was also associated with lower depression risk. Moreover, long-term consumption of vegetable oil appeared to be correlated with a decreased risk of dementia in comparison to animal oil. Nevertheless, these hypotheses did not receive confirmation in the multivariate analysis, possibly due to the presence of confounding factors that could affect outcomes through variables other than independently (51).

Besides, the univariate analysis revealed variations in food consumption between elderly individuals with and without dental restorations. Elderly individuals with missing teeth who had not received restorations exhibited lower consumption of almost all food categories, but an increased intake of sugary items, potentially leading to elevated risks of metabolic and cardiovascular diseases.

## 5 Conclusion

Oral health and dietary behavior are closely related to the susceptibility of middle-aged and elderly individuals to depression, anxiety, and dementia. The study findings revealed elderly individuals who had dental restorations were associated with a 50% lower risk of depression compared to those without restorations. Additionally, maintaining good oral hygiene practices such as brushing teeth twice or more daily was linked to a reduced risk of anxiety and dementia by 28 and 22%, respectively. Prompt treatment of tooth pain as well as pain in the cheek or jaw has shown to decrease the risk of anxiety by 21 and 31%, respectively. Furthermore, a daily intake of fresh vegetables was associated with a significant decrease in the risk of depression by 32.5%, anxiety by 50.3%, and dementia by 50%.

In China, 7.62% of elderly individuals are susceptible to depression, 36.7% to anxiety, and 7.53% to dementia. There is an urgent necessity to strengthen oral health education, improve oral hygiene, and ensure timely dental restorations to alleviate the risk of depression, anxiety, and dementia among this demographic. Addressing and managing mental health challenges in the elderly necessitate the involvement of not only psychologists but also a multidisciplinary team comprising dentists, nutritionists, and public health professionals. Additionally, the incorporation of dental and psychological care within insurance systems could potentially enhance the accessibility of resources. Further cohort studies are warranted to establish causal and temporal relationships among the variables.

## Data Availability

The original contributions presented in the study are included in the article/Supplementary material, further inquiries can be directed to the corresponding authors.
