# Supplementary material for: Modelling analysis of dietary behaviors and oral health status to assess the impact on the mental well-being of elderly individuals: a cross-sectional survey study
Source: Front Nutr. 2025 Mar 19;12:1486987. doi: 10.3389/fnut.2025.1486987 (PMC11961438; doi:10.3389/fnut.2025.1486987)
Supplement: Supplementary file 1 [file Table_1.docx]

**Supporting Information Questionnaire:**

**Modelling analysis of dietary behaviors and oral health status to assess the impact on the mental well-being of elderly individuals:** **a cross-sectional survey study**

*Chan Huang* ***^1 ,^*^*,^ ^†^,** *Mingzhu Song****^1 ,^*^*,^ ^†^**, *Xiao Wei****^1,^* ^†^***, Xingyan Wang ^1^, Honglin Dai ^1^, Zhiqiong Gou ^2^, Feiyang Chenwu ^1^, Yanqiu Jiang ^1^, Jie Wan ^1^ ,*

*Yurun Guo^1^ and Xiaoping Yu****^1 ,^*^*^**

*1. School of Preclinical Medicine & School of Nursing, Chengdu University, Chengdu, 610106, P. R. China*

*2. Department of Respiratory and thoracic Surgery, West China Tianfu Hospital, Sichuan University, Chengdu,* *625000, P. R. China*

* Corresponding authors at: School of Preclinical Medicine & School of Nursing, Chengdu University, Chengdu, 610106, P. R. China (Mingzhu Song, Email: [songmingzhu@cdu.edu.cn](mailto:songmingzhu@cdu.edu.cn), Xiaoping Yu, Email: yuxiaoping@cdu.edu.cn and Chan Huang, Email: [huangchan@cdu.edu.cn](mailto:huangchan@cdu.edu.cn))

†. Chan Huang, Mingzhu Song and Xiao Wei have contributed equally to this work and share first authorship.

1. **Oral health status,**

**Supplementary Information Questionnaire-1 (SIQ-1):**

① Number of teeth (excluding false teeth);

② Whether wearing dentures (Yes / No);

③ Number of times brushing teeth daily

0. Never brush teeth;

1. Occasionally brush teeth;

2. Once a day;

3. Twice a day;

4. Three times or more per day;

④ Whether experienced more than one toothache problem while eating in the past 6 months (Yes / No);

⑤ Pain level of the most severe toothache (1-10 scale, the higher the number, the stronger the pain);

⑥ Whether experienced facial or jaw pain more than once in the past 6 months (Yes / No);

⑦ Pain level of the most severe facial or jaw pain (1-10 scale, the higher the number, the stronger the pain).

1. **Diet and nutrition: this comprises 5 items,**

**Supplementary Information Questionnaire-2 (SIQ-2)**

1. Staple food

① Rice;

② Flour;

③ A mix of rice, flour, and others,

2. Cooking oil

① Other vegetable oils;

② Sesame oil;

③ Lard;

④ Other animal oils,

3. Intake of fresh fruits,

4. Intake of fresh vegetables,

5. Taste preference

① Light;

② Salty;

③ Sweet;

④ Spicy food;

⑤ Indeterminate taste.

**Supplementary Information Questionnaire-3 (SIQ-3)**

The survey assesses the intake of 12 types of food using a five-point scale

1. Rarely or never eat;
2. Not every day, but at least once a week;
3. Not every week, but at least once a month;
4. Not every month, but occasionally;
5. Every day/almost every day,

**(3) Depression Scale: includes 11 items:**

**Supplementary Information Questionnaire-4 (SIQ-4)**

1. Do you get upset over little things?

2. Do you have difficulty concentrating on your activities?

3. Do you feel sad or depressed?

4. Do you feel that you are becoming less useful as you get older and that everything takes effort?

5. Are you hopeful about the future?

6. Do you feel tense and scared?

7. Do you feel as happy as you were when you were young?

8. Do you feel lonely?

9. Do you feel that you cannot continue your life?

The nine items mentioned above are rated on a five-point scale ranging from 1 to 5 (1 = Always, 2 = Often, 3 = Sometimes, 4 = Rarely, 5 = Never).

10. Quality of sleep (1=better, 2=good, 3= Not bad enough , 4 =bad, 5=worse)

11. How long you sleep each day? (Hours)

**(4) Anxiety Scale: includes 7 items:**

**Supplementary Information Questionnaire-5 (SIQ-5),**

(0 = None, 1 = Several days, 2 = More than half the days, 3 = Almost every day)

1. Feel restless, worried, and irritable;

2. Unable to stop or control worry;

3. Worrying too much about various things;

4. Feel very tense and hard to relax;

5. Feel so restless that it is difficult to sit still;

6. Easily annoyed or irritated;

7. Feel like something terrible might happen.

**(5) Cognitive Function:**

**Evaluated through the use of the Community Screening Instrument for Dementia (CSI-D), which comprises seven items:**

**Supplementary Information Questionnaire-6 (SIQ-6),**

1. What do people usually use to cut paper?

2. Do apples grow on trees or in the ground?

3. Who is the current president of China?

4. What is this part called? (pointing to the elbow)

5. What is a hammer generally used for?

6. Where is the nearest store around here?

7. Please point to the window first, then the door.

**(6) General Characteristics: includes six items:**

**Supplementary Information Questionnaire-7 (SIQ-7)**

1. Age (years);

2. Gender

① Male;

② Female;

3. Ethnicity

① Han;

② Minority

4. Living situation

① Home-based care: living with family and being cared for by family;

② Community-based care: living alone supported by the community;

③ Institutional care: living in care institutions;

5. Smoking (smoking at least one cigarette daily in the past month:

① Yes; ② No

6. Drinking (drinking at least one drink weekly in the past month:

① Yes; ② No

**(7) Basic Status:** i**ncludes three items:**

**Supplementary Information Questionnaire-8 (SIQ-8)**

1. Living Conditions

① Very good;

② Good;

③ Average;

④ Not good;

⑤ Very bad;

2. Health Status

① Very good;

② Good;

③ Average;

④ Not good;

⑤ Very bad

3. Changes in Health Status over the Past Year

① Much improved;

② Somewhat improved;

③ Unchanged;

④ Somewhat worse;

⑤ Much worse
